# Development of Japanese versions of the Autoimmune Bullous Disease Quality of Life and Treatment of Autoimmune Bullous Disease Quality of Life questionnaires

**DOI:** 10.1111/1346-8138.17707

**Published:** 2025-03-20

**Authors:** Chika Tanemura, Maya Nunotani, Kyoko Kawabata, Yuki Morooka, Jun Yamagami, Risa Kakuta, Yasuko Saito, Yuichi Kurihara, Hayato Takahashi, Norito Ishii, Hiroshi Koga, Takekuni Nakama, Daisuke Hayashi, Sho Hiroyasu, Chiharu Tateishi, Daisuke Tsuruta, Dedee F. Murrell, Takashi Hashimoto

**Affiliations:** ^1^ School of Nursing Mukogawa Women's University Hyogo Japan; ^2^ Department of Dermatology Tokyo Women's Medical University Hospital Tokyo Japan; ^3^ Department of Dermatology Keio University School of Medicine Tokyo Japan; ^4^ Department of Dermatology Kurume University School of Medicine Fukuoka Japan; ^5^ Department of Dermatology Graduate School of Medicine, Osaka Metropolitan University Osaka Japan; ^6^ Department of Dermatology St George Hospital, University of New South Wales Sydney New South Wales Australia

**Keywords:** autoimmune bullous disease, Japanese versions of the questionnaires, pemphigoid, pemphigus, quality of life

## Abstract

Patients with autoimmune bullous disease have their quality of life (QOL) affected by both the disease and its treatment burden. While QOL assessment is clinically important, it is often hindered by limited time in clinical practice, highlighting the need for accurate and efficient QOL evaluation tools. However, no validated QOL questionnaires are currently available in Japan. This study evaluated the validity and reliability of the Japanese versions of the Autoimmune Bullous Disease Quality of Life (ABQOL) and Treatment of Autoimmune Bullous Disease Quality of Life (TABQOL) questionnaires, as well as their practical application in clinical settings. The original questionnaires were forward and back‐translated into Japanese by certified translators according to established guidelines, then their validity and reliability were evaluated using data from 147 patients with autoimmune bullous diseases. Validity was evaluated via confirmatory and exploratory factor analyses, cross‐cultural validation, hypothesis testing, and convergent validity. Reliability was evaluated via test–retest and internal consistency. Although confirmatory factor analysis showed a weak fit and factor structures slightly differed from the original versions, internal consistency was cross‐culturally valid. Also, the Japanese version cohort showed lower mean scores and better QOL outcomes compared with other language versions for similar cohorts. Hypothesis testing revealed a significant positive correlation between ABQOL scores and subjective disease severity; TABQOL scores were significantly correlated with steroid dosage. The mucosal subscale of the ABQOL showed a significant difference based on mucosal lesion status. Bland–Altman plots confirmed approximate agreement between the two sets of measurements: Cronbach's alpha coefficients were 0.872 for ABQOL and 0.903 for TABQOL, verifying reliability. Finally, an expert panel reviewed and agreed on the target population, timing, methods for using the scales, and considerations for scale evaluation. The Japanese versions of the ABQOL and TABQOL are expected to be implemented in clinical practice as reliable and validated tools in Japan.

## 1 INTRODUCTION

Autoimmune bullous disease, a rare and chronic disorder characterized by the formation of blisters and erosions on the skin and various mucous membranes throughout the body, is caused by autoantibodies that target proteins responsible for epithelial cell adhesion in the skin and mucosa. Thus far, advances in diagnosis and treatment have reduced the life‐threatening nature of the disease. However, relapses are frequent and often require long‐term treatment and disease management. Patients with autoimmune bullous disease experience diverse challenges to their quality of life (QOL), including physical symptoms such as pain, itching, and scarring associated with skin and mucosal lesions, as well as effects on daily life, changes in appearance, a lack of understanding from others, psychosocial impacts due to anxiety about relapses, and the need to manage long‐term treatment side effects.^1^, ^2^ Therefore, it is important for healthcare providers to assess the impact of autoimmune bullous disease on an affected patient's QOL and to engage the patient in treatment decisions as part of their daily care and management.^1^

In recent years, clinical practice has emphasized the importance of shared decision making (SDM), whereby patients and healthcare providers collaborate to make treatment decisions.^3^ SDM involves healthcare providers, patients, and families sharing information about treatment options, risks and benefits, patient values, preferences, and circumstances, along with the best available evidence.^4^ This approach is important for patients who require long‐term management and treatment, such as those with autoimmune bullous diseases. Considering the emergence of new treatment options such as rituximab, in addition to conventional therapies including oral and/or topical corticosteroids and immunosuppressive agents, SDM will play a more important role in the future management of autoimmune bullous diseases. As such, QOL assessment is clinically important; however, limited clinical time can be a significant barrier to accurate evaluation. Therefore, to facilitate SDM in the context of limited clinic time, QOL must be assessed quickly and accurately; the information must be collected in an appropriate manner and promptly communicated to patients.

Thus far, QOL assessments for patients with autoimmune bullous disease have been conducted with the MOS Short‐Form 36‐Item Health Survey (SF‐36), which measures overall health‐related quality of life (HRQOL); the Dermatology Life Quality Index (DLQI), which focuses on skin disease‐specific HRQOL; and the Skindex‐29, which also assesses HRQOL. However, these tools have shown limited sensitivity concerning the specific daily‐life impacts of blistering and mucosal lesions typical of autoimmune bullous disease, as well as the psychosocial burden of long‐term treatment with steroids, immunosuppressive drugs, and other adjuvant therapies. To address this gap, an Australian research group developed two disease‐specific QOL measures in 2013: the Autoimmune Bullous Disease Quality of Life (ABQOL) and the Treatment of Autoimmune Bullous Disease Quality of Life (TABQOL) scales.^5^, ^6^ The ABQOL explores the impact of the disease on symptoms, mucosal involvement, and psychosocial aspects, whereas the TABQOL assesses the burden associated with treatment. Translations of these scales have been developed in multiple countries.^7^, ^8^, ^9^, ^10^, ^11^, ^12^, ^13^, ^14^ However, these have not been developed in Japan. In this study, we aim to evaluate the validity, reliability, and practicality of Japanese versions of the ABQOL and TABQOL scales in clinical practice.

## 2 METHODS

### 2.1 Design

The validity and reliability of the Japanese versions of the scales were evaluated using 147 valid responses from a survey of 174 patients with various autoimmune bullous diseases; these patients were attending outpatient clinics at two expert facilities for blistering disease (Kurume University Hospital and Osaka Metropolitan University Hospital) or were members of patient associations (Japanese Pemphigus and Pemphigoid Foundation), and the data were collected cross‐sectionally from December 2022 to June 2023. Test–retest reliability was assessed using longitudinal survey data from 15 patients with pemphigus and pemphigoid; these data were obtained at another expert facility for blistering disease (Keio University Hospital).

### 2.2 Ethical approval

This study was conducted after ethical approval had been obtained from Mukogawa Women's University (No. 23‐66, No. 24‐46), Keio University (No. 20170010), and Kurume University (No. 23191). Additionally, permission to conduct clinical research was obtained from Osaka Metropolitan University (No. 2022‐0012). All participants received a written explanation of the study's purpose and methods. Those who agreed to participate indicated their consent by checking a box before completing the survey. The written instructions also explained that participation was voluntary and that choosing not to respond would not disadvantage future treatment or care.

### 2.3 Instruments or measures

#### 2.3.1 Patient recruitment

Participants were patients with various autoimmune bullous diseases attending an outpatient dermatology clinic. The exclusion criteria were (1) under 20 years old, (2) hereditary blistering diseases other than autoimmune blistering diseases, (3) cognitive decline impairing the ability to complete the questionnaire, and (4) significant physical or mental health deterioration due to disease symptoms, treatment, or other factors. Participants for the retest were selected based on stable disease severity and unchanged treatment at the time of the 3‐month follow‐up.

#### 2.3.2 Data collection method

Questionnaires were distributed either by mail or in person during the participants' outpatient visits. Completed questionnaires were returned either directly to the outpatient reception desk or in a provided return envelope, depending on the participant's preference.

#### 2.3.3 ABQOL questionnaires, and TABQOL questionnaires

The ABQOL is a scale developed to measure disease‐specific QOL in patients with autoimmune bullous disease.^5^ This scale comprises 17 items: (1) pain, (2) itching, (3) healing, (4) clothing changes, (5) bathing/showering, (6) pain (mouth), (7) gingival bleeding, (8) food avoidance, (9) embarrassment, (10) depression, (11) anxiety, (12) family/friends, (13) sexual activity, (14) relationships, (15) social life, (16) work and study, and (17) discrimination. The scale is further divided into three subscales: symptom (items 1 to 5), mucosal (items 6 to 8), and psychosocial (items 9 to 17).

The TABQOL is a scale designed to assess QOL associated with treatment burden in patients with autoimmune bullous disease.^6^ This scale comprises 17 items: (1) bruising or bleeding, (2) tolerating heat or cold, (3) medication timing, (4) number of medications, (5) feeling bloated, (6) difficulty walking, (7) clear thinking, (8) time‐consuming, (9) blood tests, (10) risk of relapse, (11) dangerous medications, (12) lethargy, (13) immunosuppression, (14) fear of getting sick, (15) nightmares, (16) holidays, and (17) financial difficulties. A three‐factor structure was identified in the original version, but factor analysis revealed that the items were loaded on two factors: item 1 was loaded on factor 3, and items 2 to 17 were loaded on factor 1. Although the nature of the single factor was strong, it was considered to contain components of the various side effects of medications, issues around taking or monitoring treatment, and concerns regarding disease side effects.^6^

Both the ABQOL and TABQOL questionnaires require responses to 17 items, rated on a scale from 0 to 3 (0, not at all/never; 1, a little/sometimes; 2, a lot/very often; 3, very much/all the time), reflecting the past week. Scores for both ABQOL and TABQOL range from 0 to 51. The scores are categorized as follows: 0–6, low impact; 7–20, medium impact; and 21–51, high impact. Higher scores indicate worse QOL.^15^

### 2.4 Process for translating the ABQOL and TABQOL into Japanese

The translation process followed the principles outlined in the Good Practice for the Translation and Cultural Adaptation Process for Patient‐Reported Outcomes Measures: Report of the International Society for Pharmacoeconomics and Outcomes Research Task Force for Translation and Cultural Adaptation.^16^

#### 2.4.1 Forward translation

The Japanese versions of the ABQOL and TABQOL were developed at Keio University with permission from the original authors. Two physicians specializing in bullous diseases, who are native Japanese speakers and fluent in English, conducted the forward translation. These physicians reviewed the clarity of the translation, the content validity of the questionnaire items, and the appropriateness of the Japanese wording. The two translations were then integrated into a single version of the scale.

#### 2.4.2 Back translation

For the back translation, a native‐English‐speaking translator, who had no access to the original English version, translated the Japanese version of the scale back into English. The back‐translated content was reviewed with the original author and two Japanese physicians specializing in bullous diseases to identify any changes, discrepancies, or differences from the original version.

#### 2.4.3 Surface validity, content validity, and cultural adaptation

The Japanese versions of the scales were pretested with several patients with autoimmune bullous disease, as well as medical office staff members and residents. Cognitive debriefing was conducted to ensure that questions in the translated versions of the scales were valid, that the content and expressions were generally understandable, and that there were no difficulties in answering the questions.

### 2.5 Data analysis

All statistical analyses were performed using SPSS Amos version 28 and SPSS version 28. Data were analyzed in accordance with the COSMIN design checklist for patient‐reported outcome measures.^17^ Data normality was confirmed using histograms and the Shapiro–Wilk test, based on a threshold of *P* < 0.001. Non‐normally distributed data were compared using Spearman's rank correlation coefficient or the Mann–Whitney *U* test.

#### 2.5.1 Item analysis

Descriptive statistics for the 147 responses were calculated to identify ceiling and floor effects. Item analysis was conducted to ensure that the percentage of missing data for each item did not exceed 10%. An item‐total correlation analysis was also performed; values between 0.3 and 0.7 were considered appropriate.^18^, ^19^

#### 2.5.2 Validity

##### Construct validity

###### Confirmatory factor analysis

Confirmatory factor analysis was conducted to verify the construct validity based on the subscale structure of the original version. Model fit was assessed using the *χ* ^2^ value, Goodness of Fit Index (GFI), Adjusted Goodness of Fit Index (AGFI), Comparative Fit Index (CFI), and Root Mean Square Error of Approximation (RMSEA). A non‐significant *χ* ^2^ value was preferred; smaller values were considered optimal. GFI, AGFI, and CFI values close to 1.00 were desirable; values above 0.90 indicated a good fit.^20^ The target RMSEA was less than 0.05; if necessary, values <0.10 were permitted.^20^ Confirmatory factor analysis for the Japanese version was conducted based on the structural validity results of the original version.

###### Exploration of factor analysis

An additional factor analysis was performed on the Japanese ABQOL data based on the goodness‐of‐fit results from the original version. TABQOL first conducted a confirmatory factor analysis of the one‐factor model, followed by an exploratory factor analysis using Japanese data to assess the goodness of fit of the Japanese version of the model.

To conduct the factor analysis, the adequacy of the sample size was confirmed using the Kaiser‐Meyer‐Olkin measure, and Bartlett's test of sphericity was performed to determine whether correlations existed between variables, ensuring that factor analysis was appropriate. Next, a scree plot was generated to identify the number of factors based on eigenvalues greater than 1. The exploratory factor analysis was repeated to examine the factor structure and identify potential subscales. Subscales were selected based on factor loadings of 0.4 or higher.^21^ If multiple factors showed high loadings, or loadings were small, subscale classification was determined by the interpretability of the data.

##### Cross‐cultural validity

In addition to verifying cultural adaptation during the translation process, we compared the factor structure, internal consistency, and mean scores of the Japanese versions of the ABQOL and TABQOL with those of other language versions.

##### Criterion‐related validity

To evaluate criterion‐related validity, we tested the hypothesis that disease severity, steroid dosage, and mucosal lesion status can serve as external criteria. We calculated correlation coefficients between these criteria and the ABQOL and TABQOL scores to assess convergent validity.

###### Hypothesis testing

The indices used for hypothesis testing were disease severity, steroid dosage, and the presence of mucosal symptoms. Since this study was conducted using a self‐administered questionnaire and mail method, disease severity was defined as the subjective severity of the patient's self‐perceived health status as recommended by the World Health Organization (WHO), on a 5‐point scale (1, very good; 2, good; 3, normal; 4, bad; 5, very bad).^22^, ^23^ Based on previous reports indicating that disease severity generally reduces QOL,^24^, ^25^, ^26^ we assumed a positive correlation between disease severity or steroid dosage and ABQOL/TABQOL scores. The steroid dosage was determined as the daily prednisolone dose per kilogram of body weight at the time of the survey; if betamethasone was used, the dose was multiplied by 6.6 and converted to the prednisolone equivalent. We also hypothesized that the ABQOL mucosal subscale scores would differ significantly between groups depending on mucosal lesion status.

###### Convergent validity

As part of hypothesis testing, we hypothesized a significant correlation between ABQOL and TABQOL because both are measures of QOL in patients with autoimmune bullous disease, therefore we tested convergent validity to confirm this relationship.

#### 2.5.3 Reliability

##### Internal consistency

Cronbach's alpha coefficients were calculated for the overall scale and each subscale. Internal consistency was considered acceptable if the alpha coefficients were 0.70 or higher.^18^, ^19^

##### Test–retest reliability

Test–retest reliability was assessed using data from the first and second surveys of 15 patients with pemphigus or pemphigoid whose disease severity and treatment remained stable over the 3 months of the previous longitudinal study. The difference and mean of the two measurements were calculated, and a Bland–Altman plot was generated to visually assess the agreement between the two measurements.

#### 2.5.4 Practicality

To consider real‐world applications of the scale in clinical practice, an expert panel was convened with six members: three physicians specializing in blistering diseases, one nurse with over 10 years of experience in blistering care, one nursing researcher familiar with scale development, and one investigator planning to conduct a scale survey. These experts were selected based on their experience using the scale.

## 3 RESULTS

### 3.1 Surface validity, content validity, and cultural adaptation

In the translated ABQOL, patients expressed difficulty in responding to item 15 (“sexual activity”) and item 17 (“discrimination”). However, item 15 was retained because it addresses a topic that may not be screenable without a questionnaire but is important for triggering potential interventions. Patients were informed that answering this item was entirely voluntary, and they agreed to leave the item. For item 17 (“discrimination by employer”), one respondent noted that housewives might be unsure whether to consider their husbands as employers. In such cases, patients were informed that selecting “Rating 0: Not relevant to this question” was acceptable or that they could respond based on their own interpretation and values. In the translated TABQOL, no specific issues were raised. The above process confirmed the validity of each translation. The Japanese versions of the ABQOL and TABQOL were finalized with the approval of the original authors.

### 3.2 Participant characteristics

Questionnaires were distributed to 174 patients and 147 were returned (84.5% response rate). The mean age of participants was 65.44 years (range 39–91 years). The disease types were as follows: pemphigus vulgaris in 48 patients (32.7%), pemphigus foliaceus in 18 patients (12.2%), bullous pemphigoid in 57 patients (38.8%), and mucous membrane pemphigoid in 15 patients (10.2%); a few patients were diagnosed with other types. A summary of participant characteristics is shown in Table 1.

**TABLE 1:** Participant characteristics.

Variable	Value *n* = 147	*n* = 15 (retest)
Age (years)		
Mean (range)	65.44 (39–91)	56.0 (24–80)
Median	67.0	58.0
Sex, *n* (%)		
Male	66 (44.9)	8 (53.3)
Female	80 (54.4)	7 (46.7)
No data	1 (0.7)	
Duration of disease (months)		
Mean (range)	84.18 (2–420)	70 (15–276)
Median	58	51
Autoimmune bollous disease, *n* (%)		
Pemphigus vulgaris	48 (32.7)	11 (73.3)
Pemphigus foliaceus	18 (12.2)	3 (20.0)
Bullous pemphigoid	57 (38.8)	1 (6.7)
Mucous membrane pemphigoid	15 (10.2)	0 (0.0)
Epidermolysis bullosa acquisita	2 (1.4)	0 (0.0)
Linear IgA bullous dermatosis	0 (0)	0 (0.0)
Others	4 (2.7)	0 (0.0)
No data	3 (2.0)	
Disease severity, *n* (%)		PDAI/BPDAI
1: Very good	28 (19)	1 point: 10 (66.7)
2: Good	45 (30.6)	2 points: 1 (6.7)
3: Normal	65 (44.2)	3 points: 2 (13.3)
4: Bad	6 (4.1)	4 points: 1 (6.7)
5: Very bad	2 (1.4)	5 points: 1 (6.7)
No data	1 (0.7)	
Skin lesion +, *n* (%)	70 (47.6)	2 (13.3)
Mucosal lesions +, *n* (%)	44 (29.9)	4 (26.7)
Treatment, *n* (%)		
Steroid medication	126 (85.7)	15 (100.0)
Mean oral dose (mg/kg/day)	0.104 (0–1)	0.16 (0.07–0.82)^a^
Immunosuppressants	35 (23.8)	9 (60.0)
Minocycline, etc.	9 (6.1)	0 (0.0)
Diaminodiphenyl sulfone	8 (5.4)	0 (0.0)
Nicotinic acid amide	10 (6.8)	0 (0.0)
Rituximab	2 (1.4)	0 (0.0)
Topical ointment therapy	61 (41.5)	0 (0.0)

Abbreviations: BPDAI, Bullous Pemphigoid Disease Area Index; PDAI, Pemphigus Disease Area Index.

^a^ Two of the 15 patients had unknown weights; the mean and range are reported for the remaining 13.

### 3.3 Japanese ABQOL and TABQOL

#### 3.3.1 ABQOL and TABQOL scores

**FIGURE 1:**
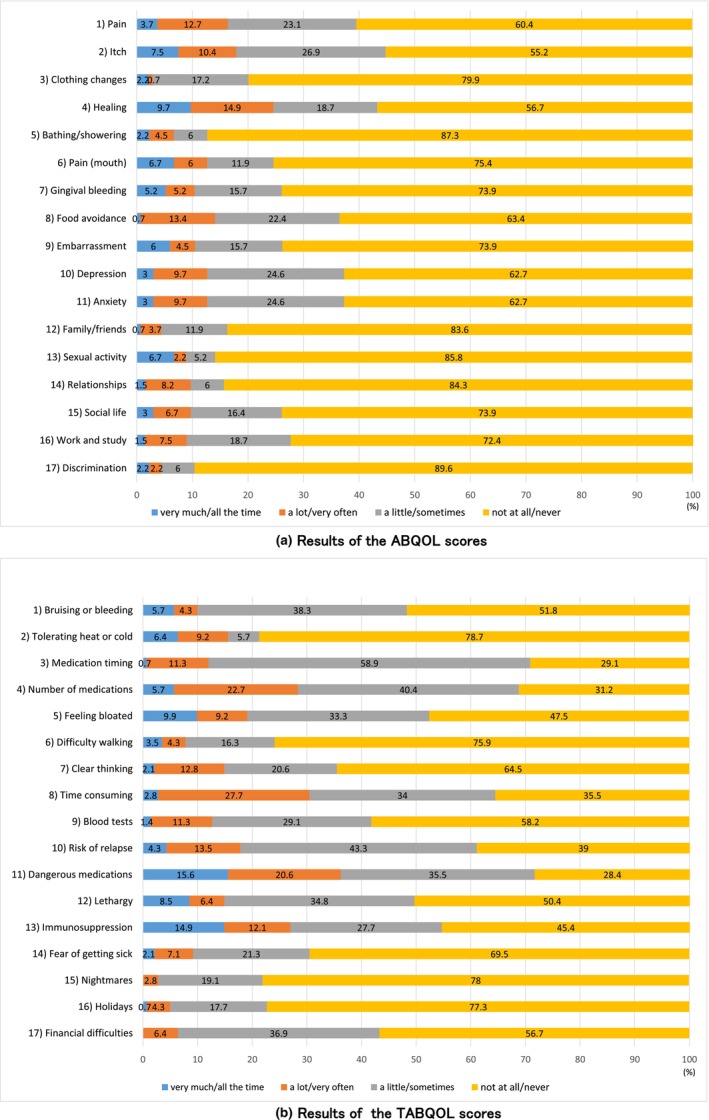
(a) Results of the ABQOL scores. The distribution of scores on 17 items of the ABQOL questionnaire, rated on a scale from 0 to 3 (0, not at all/never; 1, a little/sometimes; 2, a lot/very often; 3, very much/all the time), reflecting the past week. (b) Results of the TABQOL scores. Shows the distribution of scores on 17 items of the TABQOL questionnaire, rated on a scale from 0 to 3 (0, not at all/never; 1, a little/sometimes; 2, a lot/very often; 3, very much/all the time), reflecting the past week.

The distributions of ABQOL and TABQOL scores are shown in Figure 1a,b.

#### 3.3.2 Item analysis

The descriptive statistics, missing value analysis, and item‐total correlation results for the 147 ABQOL and TABQOL responses are shown in Table 2.

**TABLE 2:** Descriptive statistics, missing values, and item‐total correlation analysis *n* = 147.

Item No.	Description of Item	Mean	SD	Median	Minimum	Maximum	Percentile			Missing	Missing percentage	Item‐total correlation analysis
							25	50	75			
ABQOL												
1	Pain	0.60	0.851	0	0	3	0	0	1	2	1.4	0.665*
2	Itching	0.70	0.934	0	0	3	0	0	1	2	1.4	0.612*
3	Clothing changes	0.25	0.584	0	0	3	0	0	0	3	2.1	0.479*
4	Healing	0.78	1.031	0	0	3	0	0	1.25	2	1.4	0.688*
5	Bathing/showering	0.22	0.630	0	0	3	0	0	0	2	1.4	0.443*
6	Pain (mouth)	0.44	0.880	0	0	3	0	0	0.25	2	1.4	0.431*
7	Gingival bleeding	0.42	0.816	0	0	3	0	0	1	2	1.4	0.316*
8	Food avoidance	0.51	0.753	0	0	3	0	0	1	2	1.4	0.408*
9	Embarrassment	0.43	0.835	0	0	3	0	0	1	2	1.4	0.476*
10	Depression	0.53	0.792	0	0	3	0	0	1	2	1.4	0.708*
11	Anxiety	0.53	0.792	0	0	3	0	0	1	2	1.4	0.766*
12	Family/friends	0.22	0.540	0	0	3	0	0	0	2	1.4	0.515*
13	Sexual activity	0.30	0.814	0	0	3	0	0	0	9	6.5	0.554*
14	Relationships	0.27	0.673	0	0	3	0	0	0	3	2.1	0.521*
15	Social life	0.39	0.745	0	0	3	0	0	1	2	1.4	0.519*
16	Work and study	0.38	0.691	0	0	3	0	0	1	5	3.5	0.498*
17	Discrimination	0.17	0.569	0	0	3	0	0	0	8	5.8	0.394*
Total score		7.13	7.517	4	0	33	1.75	4	12			
TABQOL												
1	Bruising or bleeding	0.64	0.813	0	0	3	0	0	1	2	1.4	0.618*
2	Tolerating heat or cold	0.43	0.905	0	0	3	0	0	0	2	1.4	0.461*
3	Medication timing	0.84	0.639	0	0	3	0	1	1	2	1.4	0.382*
4	Number of medications	1.03	0.878	0	0	3	0	1	2	2	1.4	0.630*
5	Feeling bloated	0.82	0.968	0	0	3	0	1	1	2	1.4	0.652*
6	Difficulty walking	0.35	0.728	0	0	3	0	0	0	2	1.4	0.482*
7	Clear thinking	0.52	0.798	0	0	3	0	0	1	3	2.1	0.620*
8	Time‐consuming	0.98	0.866	0	0	3	0	1	2	3	2.1	0.675*
9	Blood tests	0.56	0.750	0	0	3	0	0	1	2	1.4	0.617*
10	Risk of relapse	0.83	0.819	0	0	3	0	1	1	2	1.4	0.592*
11	Dangerous medications	1.23	1.033	0	0	3	0	1	2	2	1.4	0.767*
12	Lethargy	0.73	0.917	0	0	3	0	0	1	2	1.4	0.713*
13	Immunosuppression	0.96	1.085	0	0	3	0	1	2	3	2.1	0.681*
14	Fear of getting sick	0.42	0.719	0	0	3	0	0	1	2	1.4	0.639*
15	Nightmares	0.25	0.495	0	0	2	0	0	0	2	1.4	0.525*
16	Holidays	0.28	0.577	0	0	3	0	0	0	3	2.1	0.630*
17	Financial difficulties	0.50	0.617	0	0	2	0	0	1	3	2.1	0.504*
Total score		11.38	8.669	9	0	40	5	9	16			

Abbreviations: ABQOL, Autoimmune Bullous Disease Quality of Life; TABQOL, Treatment of Autoimmune Bullous Disease Quality of Life; SD, Standard Deviation.

* Correlation coefficient significant at 1% level (two‐tailed).

##### ABQOL

Non‐normality of the data was confirmed using a histogram and the Shapiro–Wilk test (*P* < 0.001). No ceiling or floor effects were observed. Thirteen of the 147 respondents had missing values, resulting in a 91.2% valid response rate. The percentage of missing values per item ranged from 1.4% to 6.5%; the highest missing values were observed for item 13 (“sexual activity”) at 6.5%, item 17 (“discrimination”) at 5.8%, and item 16 (“work and study”) at 3.5%. The item–total correlation slightly exceeded the baseline value of 0.7 for item 10 (“depression”) (*r* = 0.708) and item 11 (“anxiety”) (*r* = 0.766).

##### TABQOL

Non‐normality of the data was confirmed using a histogram and the Shapiro–Wilk test (*P* < 0.001). No ceiling or floor effects were observed. Six of the 147 respondents had missing values, resulting in a 95.9% valid response rate. The percentage of missing values per item ranged from 1.4% to 2.1%, with 2.1% missing for items 7 (“clear thinking”), 8 (“time‐consuming”), 13 (“immunosuppression”), 16 (“holidays”), and 17 (“financial difficulties”). The item‐total correlation exceeded the baseline value of 0.7 for item 11 (“dangerous medications”) (*r* = 0.767) and item 12 (“lethargy”) (*r* = 0.713).

#### 3.3.3 Structural validity

##### ABQOL

**FIGURE 2:**
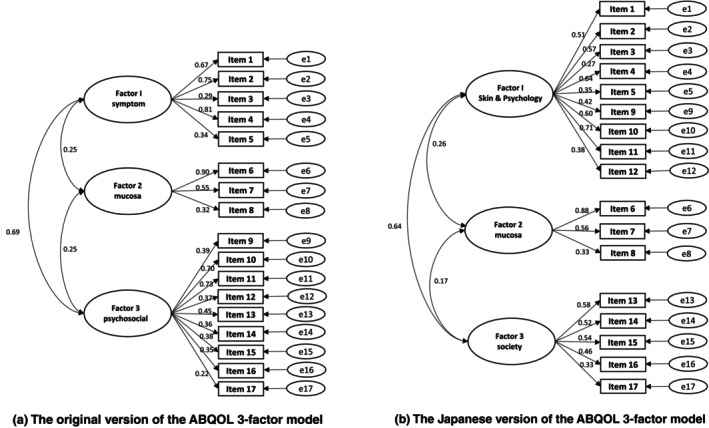
(a) The original version of the ABQOL three‐factor model. As a result of confirmatory factor analysis, the goodness of fit was weak, with *χ* ^2^ = 400.630, 116 degrees of freedom, *P* < 0.001, GFI = 0.737, AGFI = 0.653, CFI = 0.745, and RMSEA = 0.136. Factor 1, symptom; factor 2, mucosa; factor 3, psychosocial. (b) The Japanese version of the ABQOL three‐factor model. As a result of confirmatory factor analysis, the goodness‐of‐fit three‐factor model was a poor fit, with *χ* ^2^ = 384.227, 116 degrees of freedom, *P* < 0.001, GFI = 0.730, AGFI = 0.643, CFI = 0.760, and RMSEA = 0.132. Based on the item content, the factors were labeled as follows: factor 1, skin and psychology; factor 2, mucosa; factor 3, society.

A confirmatory factor analysis was initially conducted based on the subscale structure of the original version to assess the degree of fit. The goodness of fit was weak, with *χ* ^2^ = 400.630, 116 degrees of freedom, *P* < 0.001, GFI = 0.737, AGFI = 0.653, CFI = 0.745, and RMSEA = 0.136 (Figure 2a). Therefore, we examined the subscale structure of the Japanese version of the ABQOL. The Kaiser–Meyer–Olkin measure of sampling adequacy (0.817) and Bartlett's test of sphericity (*P* < 0.001) indicated that factor analysis was appropriate. Based on the scree plot and eigenvalues greater than 1, the number of factors was regarded as three. Principal component analysis with Oblimin rotation was performed in accordance with previous studies^8^, ^9^ (Table 3). Based on the item composition, the factors were named as follows: factor 1, skin and psychology; factor 2, mucosa; factor 3, society. In the Japanese version, unlike the original, psychological and social aspects were classified as separate factors, while psychological and skin‐related items were grouped into the same factor. Next, a confirmatory factor analysis was conducted on the Japanese version of the ABQOL. The results indicated a similarly poor fit, with *χ* ^2^ = 384.227, 116 degrees of freedom, *P* < 0.001, GFI = 0.730, AGFI = 0.643, CFI = 0.760, and RMSEA = 0.132. The three‐factor structural models of the Japanese version of the ABQOL are shown in Figure 2b.

**TABLE 3:** Factor analysis results for Japanese ABQOL.

Item	*n* = 134		
	Factor loadings		
	Factor 1	Factor 2	Factor 3
Factor 1: Skin and psychology			
Item 2: Itch	0.789	−0.028	−0.037
Item 5: Bathing/showering	0.763	−0.057	−0.077
Item 4: Healing	0.741	0.020	0.032
Item 1: Pain	0.708	0.351	−0.117
Item 3: Clothing changes	0.690	−0.074	−0.108
Item 12: Family/friends	0.634	−0.246	0.284
Item 9: Embarrassment	0.607	−0.040	0.073
Item 11: Anxiety	0.582	0.201	0.366
Item 10: Depression	0.567	0.192	0.325
Factor 2: Mucosa			
Item 6: Pain (mouth)	0.064	0.903	−0.092
Item 7: Gingival bleeding	−0.127	0.851	−0.049
Item 8: Food avoidance	−0.016	0.616	0.148
Factor 3: Society			
Item 15: Social life	0.030	−0.088	0.822
Item 17: Discrimination	−0.121	−0.107	0.816
Item 16: Work and study	−0.040	0.091	0.788
Item 14: Relationships	0.120	0.095	0.680
Item 13: Sexual activity	0.283	0.285	0.481
	**Factor 1**	**Factor 2**	**Factor 3**
Inter‐factor correlation factor 1	1.000		
Factor 2	0.159	1.000	
Factor 3	0.346	0.149	1.000

Abbreviations: ABQOL, Autoimmune Bullous Disease Quality of Life.

*Note*: Factor extraction method: principal component analysis. Rotation method: Oblimin rotation with Kaiser regularization. The shades of gray indicate the factor assigned based on the factor loadings.

##### TABQOL

**FIGURE 3:**
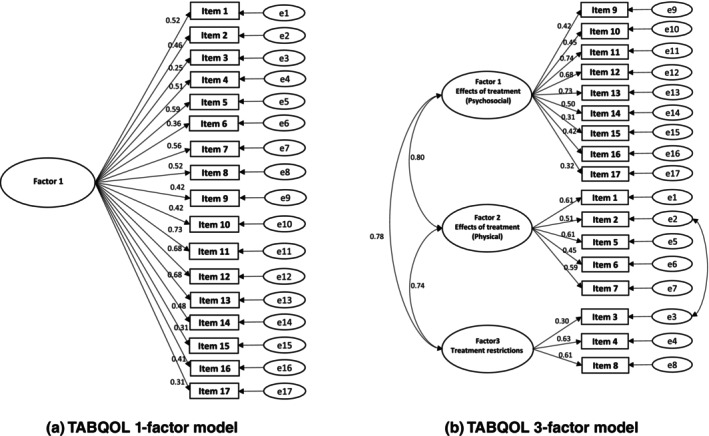
(a) TABQOL one‐factor model. As a result of confirmatory factor analysis, the goodness‐of‐fit one‐factor model was weak, with *χ* ^2^ = 252.006, 119 degrees of freedom, *P* < 0.001, GFI = 0.821, AGFI = 0.769, CFI = 0.853, and RMSEA = 0.089. (b) TABQOL three‐factor model. As a result of confirmatory factor analysis, the goodness‐of‐fit three‐factor model was not good, with *χ* ^2^ = 199.801, 115 degrees of freedom, *P* < 0.001, GFI = 0.864, AGFI = 0.819, CFI = 0.906, and RMSEA = 0.073. Based on the item content, we labeled the factors as follows: factor 1, effects of treatment (psychosocial); factor 2, effects of treatment (physical); factor 3, treatment restrictions.

A confirmatory factor analysis was initially conducted using a one‐factor structure, revealing a slightly weak fit: *χ* ^2^ = 252.006, 119 degrees of freedom, *P* < 0.001, GFI = 0.821, AGFI = 0.769, CFI = 0.853, and RMSEA = 0.089, therefore we conducted an exploratory factor analysis. The Kaiser–Meyer–Olkin measure of sampling adequacy (0.894) and Bartlett's test of sphericity (*P* < 0.001) confirmed the appropriateness of factor analysis for this data. The number of factors was determined by examining the scree plot and eigenvalues greater than 1. The scree plot indicated a one‐factor structure. However, when examining eigenvalues of 1 and above, the cumulative contribution rates for factors 1 through 4 were 40.562%, 48.496%, 55.270%, and 61.289%, respectively. Therefore, we analyzed the structure assuming between one and four factors; we found that the factor loadings and theoretically interpretable structure were optimal with a three‐factor model (Table 4). Based on the item content, we labeled the factors as follows: factor 1, effects of treatment (psychosocial); factor 2, effects of treatment (physical); factor 3, treatment restrictions. Next, a confirmatory factor analysis was conducted. The results were *χ* ^2^ = 199.801, 115 degrees of freedom, *P* < 0.001, GFI = 0.864, AGFI = 0.819, CFI = 0.906, and RMSEA = 0.073 (corrected by setting covariance for items 2 and 3 based on the adjusted index). Although the goodness of fit was not good, it was better than the fit for the one‐factor structural model. The one‐ and three‐factor structural models of the Japanese version of the TABQOL are shown in Figure 3a,b.

**TABLE 4:** Factor analysis results for Japanese TABQOL.

Item	*n* = 141		
	Factor loadings		
	Factor 1	Factor 2	Factor 3
Factor 1: Effects of treatment (psychosocial)			
Item 13: Immunosuppression	0.847	−0.156	−0.002
Item 14: Fear of getting sick	0.797	0.059	−0.147
Item 10: Risk of relapse	0.627	−0.25	0.204
Item 12: Lethargy	0.558	0.355	−0.105
Item 11: Dangerous medications	0.472	−0.042	0.404
Item 16: Holidays	0.457	0.254	0.086
Item 15: Nightmares	0.406	0.225	0.059
Item 17: Financial difficulties	0.390	0.132	0.040
Item 9: Blood tests^a^	0.315	0.023	0.314
Factor 2: Effects of treatment (physical)			
Item 6: Difficulty walking	−0.195	0.770	0.031
Item 1: Bruising or bleeding	−0.044	0.737	0.065
Item 7: Clear thinking	0.242	0.658	−0.115
Item 5: Feeling bloated	0.180	0.437	0.084
Item 2: Tolerating heat or cold^a^	−0.113	0.405	0.329
Factor 3: Treatment restrictions			
Item 4: Number of medications	−0.011	0.035	0.737
Item 8: Time‐consuming	−0.016	0.278	0.486
Item 3: Medication timing	0.033	−0.013	0.476
	**Factor 1**	**Factor 2**	**Factor 3**
Inter‐factor correlation factor 1	1.000		
Factor 2	0.663	1.000	
Factor 3	0.628	0.601	1.000

Abbreviations: TABQOL, Treatment of Autoimmune Bullous Disease Quality of Life.

*Note*: Factor extraction method: maximum‐likelihood estimation. Rotation method: Promax method with Kaiser normalization. The shades of gray indicate the factor assigned based on the factor loadings.

^a^ Classified where factor loadings are small and interpretable.

#### 3.3.4 Cross‐cultural validity

The factor structure, internal consistency, and scale scores of the other language versions are presented in Table 5. In the Japanese version of the ABQOL, a three‐factor structure was confirmed, similar to the original and Chinese versions; the structure of the mucosal factor aligned with the structure in the original version. Overall, the factor structure closely resembled that of the Chinese version. In the TABQOL, a strong single‐factor structure (similar to the original version) was initially observed; however, three factors were ultimately extracted, consistent with the versions from Chinese and Persian. Cronbach's alpha coefficients, indicating internal consistency, were similarly high across all language versions.

**TABLE 5:** Comparisons with other language versions of the ABQOL and TABQOL.

Country	Language	Sample size and characteristics	Factor structure	Cronbach's alpha	Score mean ± SD	Reference numbers
Australia (original version)	English	ABQOL				
		70 AIBDs Majority of patients in remission with immunosuppressive therapy	Three factors Factor 1 (symptoms): items 1–5 Factor 2 (mucosal): items 6–8 Factor 3 (psychosocial): items 9–17	0.84	PV 11.5 ± 5.5 BP 8.4 ± 5.5 Others 11.9 ± 8.9	[5]
		TABQOL				
		70 AIBDs Majority of patients in remission with immunosuppressive therapy	Loaded on two factors, but with a strong single‐factor nature (all but item 1 were grouped within factor 1)	0.892	Total 16.3 ± 10.3 EBA 24.5 ± 0.71 PV 17.35 ± 8.80 MMP 16.75 ± 12.6 PF 16.00 ± 7.81 BP 15.1 ± 12.38 LIGA 14.3 ± 11.15	[6]
Japan	Japanese	ABQOL				
		147 AIBDs Majority of patients in remission with immunosuppressive therapy	Three factors Factor 1 (skin and psychology): items 1–5, 9–12 Factor 2 (mucosa): items 6–8 Factor 3 (society): items 13–17	Total: 0.872 Factor 1: 0.877 Factor 2: 0.737 Factor 3: 0.820	Total 7.13 ± 7.517 PV 6.55 ± 6.624 PF 4.44 ± 8.342 BP 7.70 ± 7.551 MMP 10.14 ± 8.439 EBA 17.00 (only one person)	
		TABQOL				
		147 AIBDs Majority of patients in remission with immunosuppressive therapy	Three factors Factor 1 (psychosocial): items 9–17 Factor 2 (physical): items 1–2, 5–7 Factor 3 (treatment restrictions): items 3–4, 8 Strong single‐factor nature	Total: 0.903 Factor 1: 0.859 Factor 2: 0.784 Factor 3: 0.664	Total 11.38 ± 8.669 PV 12.62 ± 9.133 PF 10.00 ± 9.911 BP 11.41 ± 8.510 MMP 11.08 ± 6.776 EBA 13.00 (only one person)	
United States	English	ABQOL				
		39 AIBDs Clinical disease stages: including partial remission on minimal therapy (*n* = 16), relapse (*n* = 9), complete remission off therapy (*n* = 7) etc.		0.9	PV 16.4 ± 2.9 BP 10.8 ± 2.5	[7]
China	Chinese	ABQOL				
		101 AIBDs Clinical disease stages: baseline (*n* = 5), complete remission during tapering (*n* = 23), complete remission on minimal therapy (*n* = 29), complete remission off therapy (*n* = 2), control of disease activity (*n* = 30), time to control of disease activity (*n* = 8), and relapse/flare (*n* = 2) etc.	Three factors Factor 1 (symptoms): items 1–5 and 9–12 Factor 2 (mucosal): items 6 and 7 Factor 3 (psychosocial): items 8 and 13–17	0.88	PV 17.23 ± 1.35 BP 16.60 ± 2.90 Others 17.16 ± 2.90	[8]
		TABQOL				
		86 AIBDs Clinical disease stages: complete remission minimal therapy (*n* = 29), control of disease activity (*n* = 27), complete remission during tapering (*n* = 23), time to control of disease activity (*n* = 3) etc.	Three factors Factor 1: items 2, 5, 6, 7, 12, 14, 15, and 16 Factor 2: items 8–11, 13, and 17 Factor 3: items 1, 3, and 4	0.883	Total 17.43 ± 9.77 PV 17.22 ± 9.59 BP 17.74 ± 11.46 Others 17.75 ± 8.73	[9]
Poland	Polish	ABQOL				
		80 AIBDs Clinical disease stages: complete remission on treatment (*n* = 19), partial remission on treatment (*n* = 16), flare (*n* = 12), complete remission off treatment (*n* = 7) etc.		0.95	Total 16.3 ± 9.9 BP 15.7 ± 9.5 PV 17.4 ± 12.4 MMP 15.4 ± 8.7 PF 22.2 ± 9.4	[10]
		TABQOL				
		80 AIBDs Clinical disease stages: complete remission on treatment (*n* = 19), partial remission on treatment (*n* = 16), flare (*n* = 12), complete remission off treatment (*n* = 7) etc.		0.87	Total 15.3 ± 9.4 BP 14.3 ± 8.7 PV 17.1 ± 12.7 MMP 14.4 ± 7.5 PF 19.5 ± 9.0	[10]
Egypt, Tunisia	Arabic	ABQOL				
		80 AIBDs The small number of patients with severe disease		0.76	Total 16.4 ± 9.2 BP 34 (only one person) PF 19 ± 3.7	[11]
		TABQOL				
		80 AIBDs The small number of patients with severe disease		0.74	Total 21.5 ± 9.4 BP 35 (only one person) PV 21.6 ± 9.6	[11]
Iran	Persian	ABQOL				
		180 AIBDs (PV) All patients had active disease	Two factors Factor 1 (symptomatic and social): items 1, 2, 3, 5, 7, 9 and 12–17 Factor 2 (mucosal/psychological): items 4, 6, 8, 10 and 11	Total: 0.855 Factor 1: 0.918 Factor 2: 0.6	Total 29.37	[12]
		TABQOL				
		Step (1) 53 PV Step (2) 119 AIBDs Prednisolone dose (median: 10, mean ± SD: 18.133 ± 18.786)	Three factors Factor 1: items 3, 8, 9, 12, 14, 15 and 17 Factor 2: items 1, 4, 5, 6 and 7 Factor 3: items 2, 10, 11 and 13 Item 16 was not grouped within any factor.	0.804	Total 13.87 ± 7.51 EBA 24 ± 8.485 PF 20.5 ± 14.181 PV 13.24 ± 6.54	[13]
Turkey	Turkish	ABQOL				
		68 AIBDs Clinical disease stages: complete remission during tapering (*n* = 13), complete remission on minimal treatment (*n* = 14), complete remission off treatment (*n* = 11), relapse/flare (*n* = 23) etc.		0.86	Total 17.7 ± 8.94 PV 17.16 ± 8.97 PF 12.66 ± 3.05 BP 19.14 ± 10.41 EBA 21.33 ± 2.08 DH 21.8 ± 11.32	[14]
		TABQOL				
		68 AIBDs Clinical disease stages: complete remission during tapering (*n* = 13), complete remission on minimal treatment (*n* = 14), complete remission off treatment (*n* = 11), relapse/flare (*n* = 23) etc.		0.88	Total 18.78 ± 9.08 PV 18.25 ± 8.78 PF 17.5 ± 7.7 BP 19.5 ± 12.62 EBA 24 ± 2.64 DH 21.33 ± 14.29	[14]

Abbreviations: ABQOL, Autoimmune Bullous Disease Quality of Life; AIBD, autoimmune bullous disease; BP, bullous pemphigoid; DH, dermatitis herpetiformis; EBA, epidermolysis bullosa acquisita; LIGA, linear IgA bullous dermatosis; MMP, mucous membrane pemphigoid; PF, pemphigus foliaceus; PV, pemphigus vulgaris; SD, Standard Deviation; TABQOL, Treatment of Autoimmune Bullous Disease Quality of Life.

The results from the scale survey showed a mean ABQOL score of 7.13 ± 7.517 and a mean TABQOL score of 11.38 ± 8.669. The mean ABQOL and TABQOL scores indicated a medium impact on QOL. This impact was similar to mean score assessments in the original, Chinese, and Turkish versions for patients with stable disease status.

#### 3.3.5 Criterion‐related validity

Because the original ABQOL version offers better theoretical interpretation and practicality than the factor structure of the Japanese version, subsequent validation was conducted using the subscale structure from the original version.

##### Hypothesis testing

Correlations of the ABQOL and TABQOL scores with disease severity are shown in Table 6. The correlation between the total ABQOL score and severity was significant (*P* < 0.001) with *r* = 0.588 (95% confidence interval [CI] 0.461–0.692). The correlation between the total TABQOL score and disease severity was *r* = 0.257 (95% CI 0.091–0.409, *P* = 0.002). Among the three‐factor structures, only the third factor (treatment restrictions) showed a significant positive correlation, with *r* = 0.330 (95% CI 0.169–0.473, *P* < 0.001).

**TABLE 6:** Validation of external criteria.

External criteria	ABQOL				TABQOL			
	Factor 1	Factor 2	Factor 3	Total	Factor 1	Factor 2	Factor 3	Total
Disease severity								
Correlation coefficient	0.518*	0.370*	0.447*	0.588*	0.19	0.224*	0.330*	0.257
Significance probability	<0.001	<0.001	<0.001	<0.001	0.024	0.008	<0.001	0.002
95% confidence interval^a^	0.378–0.635	0.209–0.512	0.295–0.576	0.461–0.692	0.020–0.348	0.056–0.380	0.169–0.473	0.091–0.409
Steroid dosage (mg/kg/day)								
Correlation coefficient	0.264	0.141	0.225	0.260	0.390*	0.338*	0.472*	0.465*
Significance probability	0.002	0.106	0.009	0.003	<0.001	<0.001	<0.001	<0.001
95% confidence interval^a^	0.093–0.421	−0.035‐0.309	0.052–0.386	0.088–0.417	0.234–0.525	0.177–0.481	0.328–0.595	0.319–0.589
Mucosal lesion status								
Significance probability	0.048	<0.001*	0.237	0.002				

Abbreviations: ABQOL, Autoimmune Bullous Disease Quality of Life; TABQOL, Treatment of Autoimmune Bullous Disease Quality of Life.

*Note*: Standard errors are estimated using the formula proposed by Fieller, Hartley, and Pearson.

^a^ Estimation is based on Fisher's *z*‐transformation.

* Correlation coefficient is significant at the 1% level (two‐tailed).

Correlations of the ABQOL and TABQOL scores with steroid dosage are shown in Table 6. The correlation between the ABQOL total score and steroid dosage was *r* = 0.260 (95% CI 0.088–0.417, *P* = 0.003). No significant differences were detected in the overall scale or any of the subscales. The correlation between the total TABQOL score and steroid dosage was *r* = 0.465 (95% CI 0.319–0.589, *P* < 0.001). Significant positive correlations with steroid dosage were observed, both overall and across all subscales.

The Mann–Whitney *U* test results for mucosal lesion status revealed a significant difference (*P* < 0.001) only for the second factor (mucosa), indicating that the clinical symptoms and the ABQOL subscale were appropriately responsive. Additionally, patients with pemphigus foliaceus had the lowest scores on the mucosal subscale relative to patients with other forms of the disease. Mean scores on the mucosal subscale were 0.19 ± 0.544 for pemphigus foliaceus, 1.68 ± 2.154 for pemphigus vulgaris, 0.85 ± 1.350 for bullous pemphigoid, and 3.43 ± 2.409 for mucous membrane pemphigoid.

##### Convergent validity

Correlations between the ABQOL and TABQOL were calculated to assess convergent validity. A significant positive correlation was identified, with *r* = 0.566 (95% CI 0.437–0.673, *P* < 0.001).

#### 3.3.6 Internal consistency

The Cronbach's alpha coefficients for the Japanese versions of the ABQOL and TABQOL are shown in Table 5. Overall, the ABQOL and TABQOL demonstrated high internal consistency with *α* = 0.872 and *α* = 0.903, respectively. For the TABQOL, only the third factor (treatment restrictions) had an alpha coefficient below the consistency threshold (*α* = 0.664).

#### 3.3.7 Test–retest reliability

**FIGURE 4:**
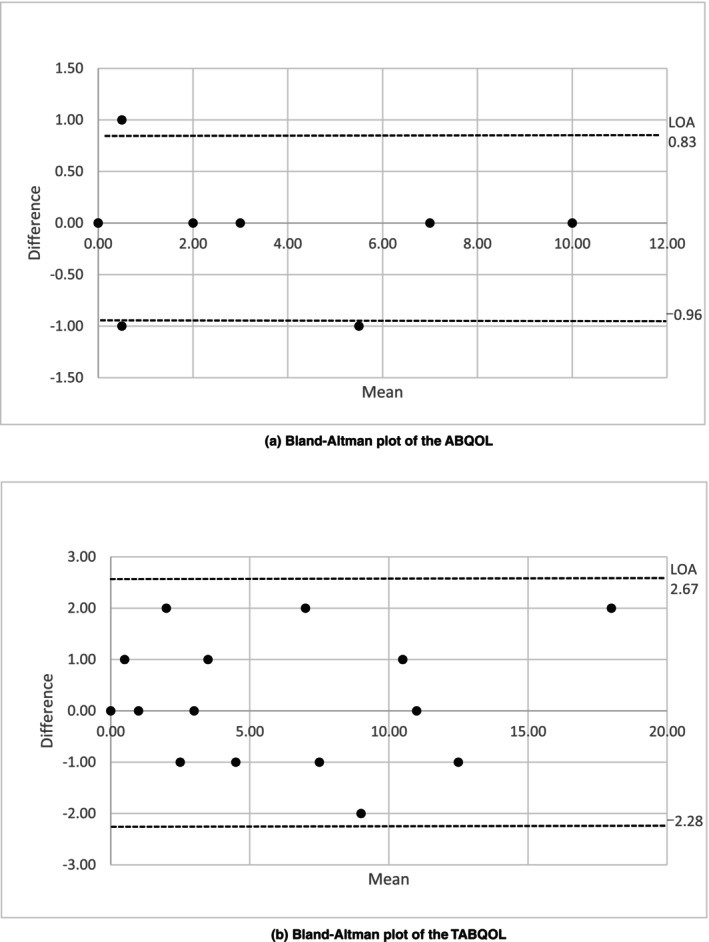
(a) Bland–Altman plot of the ABQOL. The results of the reliability test–retest. “Difference” is a variable representing the difference between two measurements. “Mean” is a variable representing the average of two measurements. “LOA” is a variable representing the limit of agreement (the 95% error range). The dotted line represents the 95% error range. The 95% error ranges were −0.96 to 0.83 for the ABQOL. (b) Bland–Altman plot of the TABQOL. The results of the reliability test–retest. “Difference” is a variable representing the difference between two measurements. “Mean” is a variable representing the average of two measurements. “LOA” is a variable representing the limit of agreement (the 95% error range). The dotted line represents the 95% error range. The 95% error ranges were −2.28 to 2.67 for the TABQOL.

Bland–Altman plots showed that the mean value for the difference between the two measurements varied, especially for TABQOL, however, it was almost within the 95% error range for both ABQOL and TABQOL (Figure 4a,b). The 95% error ranges were −0.96 to 0.83 for the ABQOL and −2.28 to 2.67 for the TABQOL.

#### 3.3.8 Practicality

The results of the expert panel discussion regarding use of these scales in clinical practice are listed in Table 7. The panel reached consensus regarding the subjects, timing, methods, and considerations for using these scales in practice.

**TABLE 7:** Results of the expert panel discussion.

Target population
Patients with autoimmune bullous disease
Patients able to complete the questionnaire
Distribution and completion of forms
Receptionists or nurses will distribute the forms
Patients with severe symptoms in the early stages of illness should be accompanied if possible
If patients have difficulty completing the form, family members or medical staff may assist
Timing
1. Soon after onset of disease (at the time of initial diagnosis)
2. After the introduction of treatment (to evaluate treatment effectiveness and side effects)
3. During steroid tapering (when there is a risk of relapse)
4. During relapse
5. When symptoms persist despite treatment
Evaluation method
1. Total score evaluation
0–6 points: Low impact on QOL
7–20 points: Moderate impact on QOL
21–51 points: High impact on QOL
Note: ABQOL scores are necessarily higher in patients with both skin and mucosal lesions
2. Subscale score evaluation
ABQOL: symptoms (items 1–5), mucosa (items 6–8), psychosocial (items 9–17)
TABQOL: Evaluated as a single factor (items 1–17)
3. Item‐by‐item evaluation
Review items with high scores (scored on a 4‐point scale from 0 to 3)
4. Trend evaluation
Compare with previous evaluation results
Feedback to patients
Review the impact on quality of life and conduct an interview with the patient
Collaborate with the patient to select future care and treatment plans based on the evaluation results
Consider collaboration with multiple healthcare professionals as needed

Abbreviations: ABQOL, Autoimmune Bullous Disease Quality of Life; QOL, quality of life; TABQOL, Treatment of Autoimmune Bullous Disease Quality of Life.

## 4 DISCUSSION

In this study, we evaluated the validity and reliability of the Japanese versions of the ABQOL and TABQOL, then assessed their real‐world applications in clinical practice. The mean steroid dosage for participants in this study was 0.1 mg/kg/day, indicating a population that remained in remission with low‐dose steroid therapy. The mean scores were 7.13 ± 7.517 for the ABQOL and 11.38 ± 8.669 for the TABQOL, both indicating a medium impact on QOL. Compared with similar patient populations in the original, Chinese, and Turkish versions,^5^, ^6^, ^9^, ^14^ the Japanese versions cohort showed lower mean scores and better QOL outcomes. These findings may reflect advancements in treatment and care over time, the practice system, and QOL considerations.

### 4.1 Validity

Confirmatory factor analysis results showed a weak fit for both the original and Japanese versions of the ABQOL. We believe that this limitation arises from evaluating the disease as a single measure, given that some disease types may not involve mucosal lesions; moreover, mucosal and skin lesions are often independent of each other. However, the inclusion of a mucosal subscale is an important feature of this QOL scale, specifically designed for autoimmune bullous diseases. Therefore, when using this scale, it is important to interpret the results with the expectation that patients with both skin and mucosal lesions inevitably will have higher scores. Conversely, patients with lesions limited to either the skin or mucosa will have lower apparent scores, therefore each item and subscale should be carefully reviewed.

Factor structure analysis revealed that the Japanese version of the ABQOL differed from the original and other language versions, such that psychological and skin‐related aspects were grouped into the same subscale. We believe this difference reflects a characteristic of Japanese culture, where skin lesion‐associated changes in appearance are closely linked to psychological well‐being. However, the expert panel suggested that—for practical use—it is more effective to apply the subscale structure of the original version, which offers a better theoretical framework. Based on the goodness‐of‐fit results comparing the Japanese and original versions of the three‐factor model, we recommend using the original three‐factor subscale structure of the ABQOL for practical applications in Japan.

The TABQOL exhibited a strong one‐factor structure, similar to the original version. However, a three‐factor structure also was identified in the Japanese version, consistent with factor analysis results from studies in Chinese and Persian versions.^9^, ^13^ Although the goodness‐of‐fit results for the Japanese version of the three‐factor model were not favorable, this structure is theoretically easier to interpret than the subscale structures of other language versions. Thus, if necessary, the TABQOL could utilize the three‐factor model developed in Japan. At this time, however, the expert panel recommends using the one‐factor model due to its practicality. Hypothesis testing revealed significant correlations between disease severity and ABQOL, as well as between steroid dosage and TABQOL. Previous studies have shown that higher disease severity is associated with lower QOL.^24^, ^25^, ^26^ Notably, even low disease activity can affect QOL,^27^ and some studies have demonstrated reduced psychosocial QOL even in the absence of active disease.^28^ The significant correlation between ABQOL and disease severity in this study may be related to the use of the patient's subjective assessment as the sole measure of severity. Future studies should explore the relationships of QOL with more objective severity indices, using international criteria such as the Pemphigus Disease Area Index (PDAI),^29^ Bullous Pemphigoid Disease Area Index (BPDAI),^30^ Mucous Membrane Pemphigoid Disease Area Index (MMPDAI),^31^ rash area, and serum antibody titers. However, ABQOL and TABQOL have been reported to correlate less with objective disease severity than DLQI and Skindex‐29.^32^ This is believed to indicate that the specific QOL of patients with autoimmune bullous disease does not depend solely on the visible severity of the disease.^28^ Therefore, it is desirable to obtain both subjective and objective measures of disease severity, and we believe it is significant that we obtained subjective severity in this study. On the other hand, the patient's self‐perceived health status, defined as subjective disease severity in this study, is positioned as part of the questions in the SF‐36 and other QOL questionnaires, therefore it should be recognized that subjective disease severity on a 5‐point scale used in this study is likely to be confounded by ABQOL and TABQOL. In addition, previous research has frequently revealed correlations between patient QOL and psychological factors such as anxiety and depression.^33^ The results of item analysis showed that item‐total correlations for item 10 (“depression”) and item 11 (“anxiety”) exceeded the established criteria. This indicates that “anxiety” and “depression” are highly associated with QOL. We believe that more multilateral investigations of severity and psychological aspects in future studies will provide greater clarity concerning the true QOL among patients with autoimmune bullous disease in Japan.

### 4.2 Reliability

The results for test–retest reliability and internal consistency were favorable. In this study, patients whose severity and treatment remained stable over the 3‐month period were carefully selected to assess reproducibility and stability. This suggests that patients' health‐related QOL is strongly related to disease severity and the nature of treatment. However, psychological changes and social factors were not considered during the 3‐month retest period, and the small sample size limits the generalizability of the findings.

### 4.3 Practicality

Finally, we propose the future use of the Japanese versions of ABQOL and TABQOL in clinical practice. The expert panel discussed and agreed that the original ABQOL subscale structure should be applied, and that the TABQOL should be used as a single‐factor measure. The panel also reached consensus regarding the subjects, timing, methods, and considerations for using these scales in practice. Given the significant correlation between ABQOL and TABQOL, indicated by their convergent validity, we recommend that both scales be used together in future clinical assessments. The findings also indicate that, in the study population in remission with low‐dose steroid therapy, the TABQOL is more responsive than the ABQOL and reflects a greater reduction in QOL associated with treatment burden. Additionally, there was a significant positive correlation between disease severity and the ABQOL. Based on these results, we propose that the two QOL scales will be used at five key time points: (1) soon after onset of disease, (2) after the introduction of treatment (to evaluate treatment effectiveness and side effects), (3) during steroid tapering (when there is a risk of relapse), (4) during relapse, and (5) when symptoms persist despite treatment. In the present study, it was not possible to calculate the minimal clinically important difference, which would indicate the implications of changes in QOL scores before and after treatment or the clinical utility of these score changes for individual patients. Despite these limitations, we hope that ABQOL and TABQOL will be adopted as tools to support SDM in clinical practice in Japan, and that more comprehensive practical guidelines will be developed.

### 4.4 Limitations

This study was based on data collected retrospectively, and some aspects of the study did not meet the recommended validity and reliability validation methods. Additional validation of the missing parts will be conducted through further research.

## CONFLICT OF INTEREST STATEMENT

Author D.T. is an editorial board member of *The Journal of Dermatology*. To minimize bias, Author D.T. was excluded from all editorial decision‐making related to the acceptance of this article for publication. There are no other conflicts of interest to declare.

## References

[1] Padniewski JJ , Shaver RL , Schultz B , Pearson DR . Patient quality of life improvement in bullous disease: a review of primary literature and considerations for the clinician. Clin Cosmet Investig Dermatol. 2022;15:27–42. 10.2147/CCID.S324331 PMC8759990 35046687

[2] Sebaratnam DF , McMillan JR , Werth VP , Murrell DF . Quality of life in patients with bullous dermatoses. Clin Dermatol. 2012;30:103–107. 22137233 10.1016/j.clindermatol.2011.03.016 PMC3883428

[3] Elwyn G . Shared decision making: what is the work? Patient Educ Couns. 2021;104:1591–1595. 33353840 10.1016/j.pec.2020.11.032

[4] Charles C , Gafni A , Whelan T . Shared decision‐making in the medical encounter: what does it mean? (or it takes at least two to tango). Soc Sci Med. 1997;44:681–692. 9032835 10.1016/s0277-9536(96)00221-3

[5] Sebaratnam DF , Hanna AM , Chee SN , Frew JW , Venugopal SS , Daniel BS , et al. Development of a quality‐of‐life instrument for autoimmune bullous disease: the autoimmune bullous disease quality‐of‐life questionnaire. JAMA Dermatol. 2013;149:1186–1191. 23925444 10.1001/jamadermatol.2013.4972

[6] Tjokrowidjaja A , Daniel BS , Frew JW , Sebaratnam DF , Hanna AM , Chee S , et al. The development and validation of the treatment of autoimmune bullous disease quality of life questionnaire, a tool to measure the quality of life impacts of treatments used in patients with autoimmune blistering disease. Br J Dermatol. 2013;169:1000–1006. 24102329 10.1111/bjd.12623

[7] Sebaratnam DF , Okawa J , Payne A , Murrell DF , Werth VP . Reliability of the autoimmune bullous disease quality of life (ABQOL) questionnaire in the USA. Qual Life Res. 2015;24:2257–2260. 25795375 10.1007/s11136-015-0965-z PMC4767525

[8] Yang B , Chen G , Yang Q , Yan X , Zhang Z , Murrell DF , et al. Reliability and validity of the Chinese version of the autoimmune bullous disease quality of life (ABQOL) questionnaire. Health Qual Life Outcomes. 2017;15:31. 28153023 10.1186/s12955-017-0594-z PMC5290597

[9] Chen G , Yang B , Zhang Z , Yang Q , Yan X , Murrell DF , et al. Chinese version of the treatment of autoimmune bullous disease quality of life questionnaire: reliability and validity. Indian J Dermatol Venereol Leprol. 2018;84:431–436. 28485307 10.4103/ijdvl.IJDVL_538_16

[10] Kalinska‐Bienias A , Jakubowska B , Kowalewski C , Murrell DF , Wozniak K . Measuring of quality of life in autoimmune blistering disorders in Poland. Validation of disease‐specific autoimmune bullous disease quality of life (ABQOL) and the treatment autoimmune bullous disease quality of life (TABQOL) questionnaires. Adv Med Sci. 2017;62:92–96. 28208086 10.1016/j.advms.2016.07.002

[11] Saleh MA , Zaraa I , Doss N , Saleh NA , Murrell DF . Assessment of the quality of life of Egyptian and Tunisian autoimmune bullous disease patients using an Arabic version of the autoimmune bullous disease quality of life and the treatment of autoimmune bullous disease quality of life questionnaires. An Bras Dermatol. 2019;94:399–404. 31644610 10.1590/abd1806-4841.20197198 PMC7007032

[12] Teimourpour A , Hedayat K , Salarvand F , Ghandi N , Ghiasi M , Mahmoudi H , et al. Autoimmune bullous disease quality of life (ABQOL) questionnaire: validation of the translated Persian version in pemphigus vulgaris. Int J Womens Dermatol. 2020;6:306–310. 33015292 10.1016/j.ijwd.2020.03.043 PMC7522916

[13] Behkar A , Garmaroudi G , Nasimi M , Yousefi S , Khosravi H , Kianfar N , et al. Assessing quality of life in patients with autoimmune bullous diseases using the Persian version of treatment of autoimmune bullous disease quality of life questionnaire finds similar effects in women as men. Int J Womens Dermatol. 2022;8:e004. 35620025 10.1097/JW9.0000000000000004 PMC9112387

[14] Bilgic‐Temel A , Irican C , Uzun S , Feng GYH , Murrell DF , Akman‐Karakas A . Quality of life in Turkish patients with autoimmune blistering diseases: reliability and validity of the autoimmune bullous disease quality of life and the treatment of autoimmune bullous disease quality of life questionnaires. Turk Dermatoloji Dergisis. 2019;13:83–90.

[15] Wang EQ , Radjenovic M , Castrillón MA , Feng GHY , Murrell DF . The effect of autoimmune blistering diseases on work productivity. J Eur Acad Dermatol Venereol. 2018;32:1959–1966. 29730897 10.1111/jdv.15062

[16] Wild D , Grove A , Martin M , Eremenco S , McElroy S , Verjee‐Lorenz A , et al. Principles of good practice for the translation and cultural adaptation process for patient‐reported outcomes (PRO) measures: report of the ISPOR task force for translation and cultural adaptation. Value Health. 2005;8:94–104. 15804318 10.1111/j.1524-4733.2005.04054.x

[17] Mokkink LB , de Vet HCW , Prinsen CAC , Patrick DL , Alonso J , Bouter LM , et al. COSMIN risk of bias checklist for systematic reviews of patient‐reported outcome measures. Qual Life Res. 2018;27:1171–1179. 29260445 10.1007/s11136-017-1765-4 PMC5891552

[18] Streiner D , Norman G , Cairney J . Health measurement scales, a practical guide to their development and use. 5th ed. Oxford, England: Oxford University Press; 2015.

[19] Kihara M , Kaji M , Kihara M . Theory and Application of Medical Measurement Scales, From Validity and Reliability to G‐Theory and Item Response Theory. 1st ed. Tokyo: Medical Science International; 2016.

[20] Oshio A . Chain of causes and effects. In: Oshio A , editor. Covariance structure analysis for beginners, path analysis by Amos. 2nd ed. Tokyo: Tokyo Tosho; 2020. p. 91–124.

[21] Oshio A . Mastering factor analysis. In: Oshio A , editor. Psychological and survey data analysis with SPSS and AMOS, up to factor analysis and covariance structure analysis. 3rd ed. Tokyo: Tokyo Tosho; 2020. p. 154–157.

[22] de Bruin A , Picavet HS , Nossikov A . Health interview surveys. Towards international harmonization of methods and instruments. WHO Reg Publ Eur Ser. 1996;58(i‐xiii):1–161. 8857196

[23] Jylhä M . What is self‐rated health and why does it predict mortality? Towards a unified conceptual model. Soc Sci Med. 2009;69:307–316. 19520474 10.1016/j.socscimed.2009.05.013

[24] Paradisi A , Sampogna F , Di Pietro C , Cianchini G , Didona B , Ferri R , et al. Quality‐of‐life assessment in patients with pemphigus using a minimum set of evaluation tools. J Am Acad Dermatol. 2009;60:261–269. 19004524 10.1016/j.jaad.2008.09.014

[25] Tabolli S , Mozzetta A , Antinone V , Alfani S , Cianchini G , Abeni D . The health impact of pemphigus vulgaris and pemphigus foliaceus assessed using the medical outcomes study 36‐item short form health survey questionnaire. Br J Dermatol. 2008;158:1029–1034. 18294312 10.1111/j.1365-2133.2008.08481.x

[26] Patsatsi A , Kokolios M , Kyriakou A , Lamprou F , Stylianidou D , Tsapas A , et al. Quality of life in Greek patients with autoimmune bullous diseases assessed with ABQOL and TABQOL indexes. Acta Derm Venereol. 2017;97:1145–1147. 28660281 10.2340/00015555-2737

[27] Bax CE , Ravishankar A , Yan D , Concha J , Kushner CJ , Zamalin D , et al. Identifying the required degree of disease clearance to improve quality of life in pemphigus vulgaris. Br J Dermatol. 2021;184:573–575. 33090460 10.1111/bjd.19625

[28] Tabolli S , Pagliarello C , Paradisi A , Cianchini G , Giannantoni P , Abeni D . Burden of disease during quiescent periods in patients with pemphigus. Br J Dermatol. 2014;170:1087–1091. 24428431 10.1111/bjd.12836

[29] Rosenbach M , Murrell DF , Bystryn JC , Dulay S , Dick S , Fakharzadeh S , et al. Reliability and convergent validity of two outcome instruments for pemphigus. J Invest Dermatol. 2009;129:2404–2410. 19357707 10.1038/jid.2009.72 PMC3010359

[30] Murrell DF , Daniel BS , Joly P , Borradori L , Amagai M , Hashimoto T , et al. Definitions and outcome measures for bullous pemphigoid: recommendations by an international panel of experts. J Am Acad Dermatol. 2012;66:479–485. 22056920 10.1016/j.jaad.2011.06.032 PMC3883429

[31] Murrell DF , Marinovic B , Caux F , Prost C , Ahmed R , Wozniak K , et al. Definitions and outcome measures for mucous membrane pemphigoid: commendations of an international panel of experts. J Am Acad Dermatol. 2015;72:168–174. 25443626 10.1016/j.jaad.2014.08.024

[32] Ferries L , Gillibert A , Duvert‐Lehembre S , Corbaux C , Alexandre M , Prost‐Squarcioni C , et al. Sensitivity to change and correlation between the autoimmune bullous disease quality‐of‐life questionnaires ABQOL and TABQOL, and objective severity scores. Br J Dermatol. 2020;183:944–945. 32374931 10.1111/bjd.19173

[33] Riopelle A , Lake E . Bullous dermatoses and quality of life: a summary of tools to assess psychosocial health. Cutis. 2022;109:E14–9. 10.12788/cutis.0439 35180062

